# CT-guided biopsies of unspecified suspect intrahepatic lesions: pre-procedure Lipiodol-marking improves the biopsy success rate

**DOI:** 10.2478/raon-2023-0024

**Published:** 2023-06-21

**Authors:** Marcel Christian Langenbach, Thomas Joseph Vogl, Amelie Buchinger, Katrin Eichler, Jan-Erik Scholtz, Renate Hammerstingl, Tatjana Gruber-Rouh

**Affiliations:** Institute for Diagnostic and Interventional Radiology, University Hospital Frankfurt, Goethe University Frankfurt, Frankfurt, Germany; Cardiovascular Imaging Research Center, Department of Radiology, Massachusetts General Hospital, Harvard Medical School, Boston, MA, USA

**Keywords:** ethiodized oil, liver, biopsy, X-ray computed tomography

## Abstract

**Background:**

While computed tomography (CT)-guided liver biopsies are commonly performed using unenhanced images, contrast-enhanced images are beneficial for challenging puncture pathways and lesion locations. This study aimed to evaluate the accuracy of CT-guided biopsies for intrahepatic lesions using unenhanced, intravenous (IV)-enhanced, or intra-arterial Lipiodol-marked CT for lesion marking.

**Patients and methods:**

Six-hundred-seven patients (men: 358 [59.0%], mean age 61 years; SD ±12.04) with suspect hepatic lesions and CT-guided liver biopsies were retrospectively evaluated. Successful biopsies were histopathological findings other than typical liver tissue or non-specific findings. Data was ascertained regarding the use of contrast medium for the biopsy-planning CT, unenhanced (group 1) *vs.* Lipiodol (group 2) *vs.* IV contrast (group 3). Technical success and influencing factors were insulated. Complications were noted. The results were analyzed using the Wilcoxon-Man-Whitney t-test, Chi-square test, and Spearman-Rho.

**Results:**

Overall lesion hitting rate was 73.1%, with significantly better rates using Lipiodol-marked lesions (79.3%) compared to group 1 (73.8%) and group 3 (65.2%) (p = 0.037). Smaller lesions (<20 mm diameter) benefited significantly from Lipiodol-marking with 71.2% successful biopsy rate compared to group 1 (65.5%) and group 3 (47.7%) (p = 0.021). Liver cirrhosis (p = 0.94) and entity of parenchymal lesions (p = 0.78) had no impact on the hitting rate between the groups. No major complications occurred during the interventions.

**Conclusions:**

Pre-biopsy Lipiodol marking of suspect hepatic lesions significantly increases the lesion-hitting rate and is especially beneficial for biopsy of smaller targets below 20 mm diameter. Further, Lipiodol marking is superior to IV contrast for non-visible lesions in unenhanced CT. Target lesion entity has no impact on the hitting rate.

## Introduction

Suspicious hepatic lesions are often detected incidentally or during staging and remain suspect despite the use of various diagnostic imaging methods. However, accurate diagnosis is crucial in the majority of suspect cases, especially in patients with known or suspected malignant disease.^1,2^ Thus, biopsies of suspicious intrahepatic lesions are an established procedure commonly performed in the clinical routine, with several options for image guidance available.

Therefore, various guiding procedures have been established for liver biopsies with good success rates providing reliable histopathological results. Ultrasound-guided biopsy is the primary option for all hepatic lesions, widely available, and allows for a biopsy performed under continuous dynamic guidance.^3,4^ However, the applicability of ultrasound is limited by the location of the lesion (*e.g.*, infra-diaphragmatic, deep in the liver) and interference with other structures, especially ribs or intestinal gas. In challenging cases with uncertain identification of the target lesions by ultrasound, magnetic resonance imaging (MRI)- or computed tomography (CT)-guidance may be beneficial for a safe and reliable biopsy.^5,6,7^

MRI guidance is technically feasible but uncommon for biopsy hepatic lesions due to high cost, time-consuming, restricted availability, and motion artifacts. The clinical applicability is commonly limited to other, more steady regions like the prostate or breast.^8^

In addition to ultrasound-guided biopsy, CT-guided biopsy is an integral part of routine clinical diagnostics. In most cases, the target liver lesions are visible in unenhanced CT images, commonly acquired for biopsy planning. Especially with the knowledge of a previous contrast-enhanced CT or MRI in which the lesions were identified and stated as suspect.^9^ Nevertheless, some hepatic lesions are challenging to delaminate in an unenhanced CT, particularly in altered liver parenchyma like hepatic cirrhosis. Also, lesions with a deep parenchymal position or with small size are challenging to detect.^10,11^ Contrast-enhanced CT images are commonly used to overcome this limitation with two options available; application of intravenous (IV) contrast medium during the examination or a previous lesion marking using Lipiodol as a contrast agent.^12,13,14^ Intravenous contrast is promptly washed out of the lesion and liver parenchyma and may not allow sufficient differentiation during the entire examination.^15,16^ In comparison, Lipiodol is interventionally administered intra-arterial during angiography and provides a long-lasting uptake (Lipiodol washout half-life 29 to 55 days^17^) in the lesion or perilesional altered tissue during the complete intervention.^12,18^

A novel approach uses fusion imaging, ultrasound, or real-time fluoroscopy synchronized with previously acquired contrast-enhanced CT or MRI images to delineate the lesion during the intervention. This technique requires the newest equipment accompanied by high costs and is limited to a few centers only.^19^

This study aimed to evaluate the interventional performance of CT-guided biopsies of suspicious intrahepatic lesions performed using unenhanced CT, intravenous contrast agent, or prior interventional lesion marking with Lipiodol for image guidance by analysis of the accuracy and potential influencing factors.

## Patients and methods

### Patient series and study setting

**FIGURE 1.**
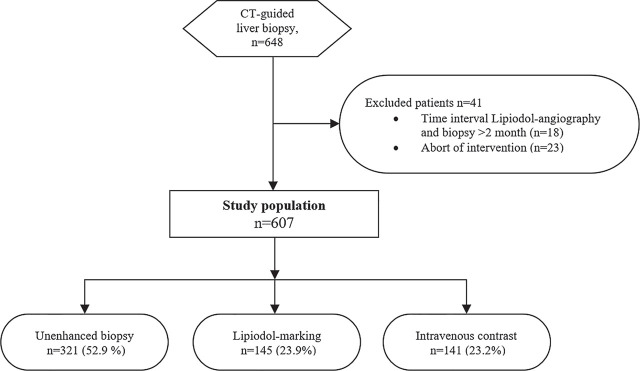
The Standards for the Reporting of Diagnostic Accuracy studies (STARD) flow diagram for a study of 607 patients undergoing CT-guided liver biopsy.

The institutional review board (IRB) approved this retrospective cohort study with a waiver for written informed consent. Inclusion criteria were as follows: unclear and suspect hepatic lesion, CT-guided biopsy of the hepatic lesion, unenhanced, IV contrast or previous Lipiodol-angiography for lesion marking, age 18 and above. All patients who met the inclusion criteria (Figure 1) were included. We excluded all patients with a time interval between CT-guided biopsy and Lipiodol-angiography of more than two months (n = 18) or an abort of the CT intervention during the examination due to lack of lesion visibility or sufficient biopsy window (n = 23).

### CT-guided biopsy

**FIGURE 2.**
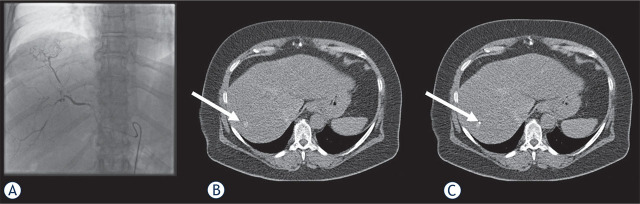
The decision for a biopsy of the suspicious hepatic lesion was made for the same male patient. Because of the small lesion size and isodensity in the unenhanced CT, a prior intra-arterial Lipiodol-marking **(A)** was performed. Directly after the intervention **(B)** and during the CT intervention **(C)** (white arrow) was well displayable, and the biopsy was successful, revealing a hepatocellular carcinoma.

**FIGURE 3.**
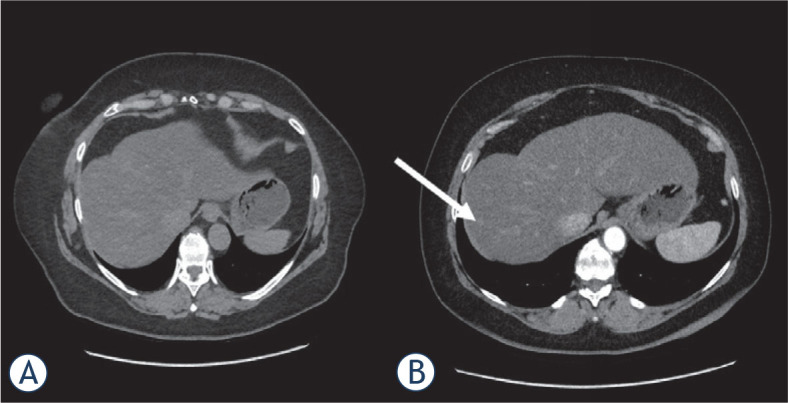
A male patient with a suspect and unclear hepatic lesion with 6 mm in segment 7. While not visible in the unenhanced CT **(A)**, the lesion impressed arterial hypervascularized after intravenous contrast medium application **(B)** (white arrow).

Data were evaluated for the whole study population, and a comparison of the three groups, unenhanced (group 1), Lipiodol enhanced (group 2), and IV contrast medium enhanced (group 3) was performed. Only patients who were not eligible for an ultrasound-guided biopsy or after an unsuccessful ultrasound-guided biopsy were referred to the radiology department for a CT-guided intervention. The interdisciplinary tumor board made the decision to biopsy. The enhancement protocol was selected at the time of the decision for biopsy based on several parameters and experience. Patients with lesions expected to be displayed well in unenhanced CT, either by size, location, or based on previous imaging, were planned for an unenhanced biopsy. A contrast protocol was determined in patients with lesions that might be more challenging to display in unenhanced CT. The decision for contrast protocol, Lipiodol (Example given in Figure 2) *vs.* IV contrast medium (*e.g.*, Figure 3), was also based on the lesions’ location and parameters (*e.g.*, size, architecture with visible changes in the liver parenchyma). Small lesions and lesions with a challenging location were more likely marked with Lipiodol due to a more long-lasting uptake which enables a secure identification during the whole examination, while IV contrast might have been washed out. In clinical practice, more intravenous contrast applications were performed in the early years of the study period. In the last years, more patients were examined using a previous intra-arterial enhancement by Lipiodol based on good local experience. The study population and the three groups were subdivided by lesion size and occurrence of liver cirrhosis.

All patients received a transcutaneous CT-guided biopsy of the hepatic lesion using the same 128-multislice CT scanner [SOMATOM Definition AS, Siemens Healthineers] for image acquisition. The interventions were performed by interventional radiologists with more than five years of experience in CT-guided interventions. The obtained core biopsies were histopathologically analyzed by the local Institute for Pathology. Successful biopsy was defined as histopathological findings other than typical liver tissue or non-specific findings. Based on our experience, Lipiodol does not affect the analysis of the specimen.

Before the examination, the coagulation profile was checked. Patients who were planned for an application of IV contrast medium were screened for contraindications, *e.g.*, reduced renal function or hyperthyroidism. The use of oral anticoagulants was prohibited based on national guidelines. No changes to institutional guidelines (standard operating procedures) were made. If patients were planned for IV contrast, 60 ml of contrast agent [Imeron® 350, Bracco] were administered with an application rate of 3–5 mL/s. We used a fixed delay of 60 s until the first planning scan was initiated.

After the patients received the first planning scan, the access path was planned using a standard 3D-CT workstation [syngo.via, Siemens Healthineers] without software modifications. In the case of multiple lesions with a similar suspect appearance, the lesion with the best reachability was defined as the target. Based on the biopsy planning, the cutaneous entry point was marked. After introducing the coaxial needle, the lesion approach was performed under image guidance. A coaxial approach was used for CT-guided intervention. This approach is characterized by combining two needles: a puncture sheath [17 G, Puncture Sheath, Somatex® Medical Technologies, Germany] and a biopsy handy [18 G, Biopsy Handy, Somatex® Medical Technologies, Germany]. The sheath - a thicker, shorter needle (5cm, 10 cm, or 15 cm) is inserted down to the anterior edge of the lesion. Then, the biopsy handy - a thinner, longer needle (10 cm, 15 cm, or 20 cm) is introduced through the sheath. Finally, the biopsy needle is inserted 2 cm into the edge of the lesion for tissue sampling. Three or four samples can be taken using the thinner biopsy needle. After the examination, additional CT single shots were performed to rule out immediate complications like bleeding or pneumothorax. Afterward, the patients had bed rest for 2 hours, a laboratory check of the blood parameters, and an ultrasound follow-up after 6 hours to rule out potential complications such as capsular hematoma or any other signs of bleeding.

### Angiography

In patients with a prior Lipiodol-marking, the angiography was performed at a minimum interval of 12 hours before the biopsy. The angiographic approach was according to our standard procedure for hepatic interventions (Figure 2A).^12^ The same team of interventional radiologists with over five years of experience conducted all interventions. The angiographic approach was through the femoral artery in all cases. After the introduction of the catheter [Boston Scientific], contrast medium iomeprol [Imeron®, Bracco] was used to display the abdominal and hepatic vessels. Following this, an overview angiography was performed. The superior mesenteric artery and celiac trunk were selectively catheterized, including an indirect portography to demonstrate vascular anatomy, ensure the portal vein's patency, and rule out portal vein thrombosis. Then, super-selective catheterization of the segmental and subsegmental hepatic branches was performed using a microcatheter [Progreat®, Terumo] for approach and precise placement. Lipiodol [Lipiodol Ultra-Fluid®, Guerbet] was administered as a tumor visualizer and transient embolic agent until stasis with a maximum amount of 15 ml. After the procedure, all patients were transferred to our ward for a minimum of 24-hour surveillance.

### Data analysis

Patient data were retrieved from the electronic patient record (Orbis, Dedalus Healthcare), imaging data from the local radiology information system (RIS, GE Healthcare), and picture archiving and communication system (PACS, GE Healthcare). We included patients who matched the inclusion criteria and received a CT-guided biopsy between 01/2010 and 12/2018.

Demographic data, underlying hepatic diseases, and liver cirrhosis were analyzed. Based on the CT images, lesion diameter, number of lesions, and lesion localization were noted. In patients with a prior Lipiodol angiography, the Lipiodol enhancement of the lesions was evaluated (visible or non-visible). During the CT-guided intervention, the puncture path (lateral-intercostal, anterolateral, anterior-subcostal, dorsolateral), length cutis-to-lesion, and liver capsule-to-lesion were measured. Dose-length-product (DLP) and the number of images of the entire examination, which includes the planning scan and the post-procedure scan, as well as for puncture sequence, including scans for needle placement, positioning, and sample acquisition, were noted and compared. This differentiation was made to achieve optimal comparability, as the differences in radiation exposure are caused by the required placement and positioning scans. For the angiography, the dose-area-product (DAB) was noted. The major endpoint of this study was the evaluation of the hitting rate, defined by histopathological findings other than healthy liver tissue. Successful biopsy findings were defined as follows: hemangioma, focal nodular hyperplasia, regenerative nodule, granuloma, adenoma, necrosis, and all types of malignancies. Unsuccessful biopsies were defined as healthy liver tissue, steatosis, inflammatory disease, fibrosis, and toxic damage.

### Statistics

IBM SPSS statistics version 24 (IBM) was used for statistical analysis. For continuous data, median, mean, standard deviation, minimum, maximum, and range were calculated. For all continuous data, results were calculated for the whole study group and the three cohorts. The Chi-square test was used to test for differences in the successful biopsies between the three cohorts. In the first step, the whole study population was analyzed. In the second step, different subgroups (*e.g.*, lesions < 20 mm, liver cirrhosis) were compared in detail.

Further, the Chi-square test was used to check for differences in the distribution of attributes within the groups. Correlations were calculated according to Spearman-Rho. P-values less than 0.05 was considered statistically significant. A logistic regression analysis was performed to screen for potential factors within the demographic factors influencing the lesion hitting rate.

### Complications

Patient records were also screened for complications, during the intervention or within the 24-hour surveillance. Complications were rated according to the severity score based on SIR's latest classification: 0 = no complications, 1 = mild adverse events (no/non-substantial therapy required), 2 = moderate adverse event (substantial treatment required), 3 = severe adverse events (escalation of care), 4 = life-threatening or disabling event, 5 = patient death. Complication score was evaluated for the whole study population and the subgroups.^20^

## Results

### Study population

Six hundred-seven patients, 358 men (59%) and 249 women (41%) (mean age 61 years; SD ± 12.04; range 22–86 years) with unclear suspect liver lesions, were retrospectively evaluated. In 321 patients (52.9%), the biopsy was obtained with unenhanced imaging, 145 lesions were prior marked with Lipiodol (23.9%), and 141 patients received IV contrast for the intervention (23.2%) (Figure 1). Liver cirrhosis was histopathologically diagnosed in 188 patients (33.0%). Patient characteristics are displayed in detail in Table 1.

**TABLE 1.** Overview of patient characteristics. P-values based on the Chi-square test indicate only a difference in the distribution of liver cirrhosis in comparison of the three enhancement groups

**Characteristic**	**Overall**	**Unenhanced biopsy**	**Lipiodol-enhanced**	**i.v. contrast-enhanced**	**p-value**
**Age, y (SD)**	61.0 ± 12.0	61.9 ± 11.95	60.4 ± 11.12	59.6 ± 13.04	0.228
**Gender, n (%)**					0.134
Male	358 (59.0)	177 (55.1)	108 (74.5)	73 (51.8)	
Female	249 (41.0)	144 (44.9)	37 (25.5)	68 (48.2)	
**Hepatic disease, n (%)**					0.354
HCV	101 (16.6)	34 (10.6)	53 (36.6)	14 (9.9)	
HBV	28 (4.6)	6 (1.9)	17 (11.7)	5 (3.5)	
HCV+HBV	8 (1.3)	3 (0.9)	3 (2.1)	2 (1.4)	
NASH	34 (5.6)	12 (3.7)	17 (11.7)	5 (3.5)	
Toxic damage	48 (7.9)	20 (6.2)	16 (11.0)	12 (8.5)	
Others	53 (8.7)	26 (8.1)	19 (13.1)	8 (5.7)	
No disease	335 (55.2)	220 (68.5)	20 (13.8)	95 (67.4)	
**Cirrhosis**	188 (33.0)	59 (19.2)	100 (71.9)	29 (3.6)	0.013
**Intrahepatic lesions, n (%)**					0.541
1 lesion	297 (48.9)	164 (51.1)	68 (46.9)	65 (46.1)	
2 lesions	116 (19.1)	45 (14.0)	37 (25.5)	34 (24.1)	
3 or more lesions	194 (32.0)	112 (34.9)	40 (27.6)	42 (29.8)	
**Lesion size, mm (SD)**	29.3 ± 18.0	30.1 ± 18.1	25.9 ± 17.4	29.8 ± 18.7	0.182

Results are presented as mean and standard deviation or number (%)

HBV = Hepatitis-B-Virus; HCV = Hepatitis-C-Virus; i.v. – intravenous; NASH – Non-alcoholic steato hepatitis; SD = Standard deviation

Most patients had a known hepatic disease (n = 310, 51.1%). The most common disease was hepatitis C in 101 cases (16.6%). Other more frequent preexisting hepatic diseases were toxic liver damage (n = 48, 7.9%), non-alcoholic steatohepatitis (NASH) (n = 34, 5.6%), and hepatitis B infection (n = 28, 4.6%). No pre-existing hepatic disease was reported in 297 patients (48.9%).

### Lesion characteristics

Based on patient medical records and the planning CT scan for the biopsy, 297 patients (48.9%) had a solitary suspect hepatic lesion, while 310 (51.1%) had two or more lesions. The target lesions were localized in all liver segments, with the majority found in liver segments 4 and 6 (each n = 112, 18.5%), and no differences according to the Chi-square test were seen between the three groups (p = 0.052). The mean lesion size was 29 ± 18 mm (range 4–116 mm). Target lesion smaller than 20 mm was seen in 229 patients (31.5%), and lesion smaller than 10 mm was seen in 38 patients (6.3%) (Table 1).

### Biopsy

Access path was in 335 cases (55.2%) from lateral-intercostal, in 224 patients (36.9%) from anterior-subcostal, in 44 cases (7.2%) from anterior-lateral subcostal and in 4 cases (0.7%) from dorsal-lateral intercostal. The mean access path length from cutis to the lesion was 83 mm (± 26 mm), and within the liver from capsule to lesion, 43 mm (± 26 mm). Per patient, 2.6 probes were obtained in mean (± 1.3), equal across all groups assessed by the Chi-square test (p = 0.307) (Table 2). Regarding the visual aspect, all lesions previously marked in the angiography were seen in the biopsy-CT, either by lesion enhancement or circular enhancement of the surrounding tissue.

**TABLE 2.** Biopsy characteristics overall and for the three enhancement groups. A Chi-square test was performed to test for differences between the groups. Significance is indicated in bold

**Characteristic**	**Overall**	**Unenhanced biopsy**	**Lipiodol-enhanced**	**i.v. contrast-enhanced**	**p-value**
**Biopsy access path**					0.718
Lateral-intercostal	335 (55.2)	162 (50.5)	94 (64.8)	79 (56.0)	
Anterior-subcostal	224 (36.9)	25 (7.8)	9 (6.2)	10 (7.1)	
Anterior-lateral	44 (7.2)	132 (41.1)	42 (29.0)	50 (35.5)	
Dorsal-lateral	4 (0.7)	2 (0.6)	0	2 (1.4)	
**Segment localization**					0.052
**1**	6 (1.0)	2 (0.6)	2 (1.4)	2 (1.4)	
2	54 (8.9)	29 (9.0)	14 (9.7)	11 (7.8)	
3	41 (6.8)	23 (7.2)	8 (5.5)	10 (7.1)	
4	112 (18.5)	66 (20.6)	23 (15.9)	23 (16.3)	
5	95 (15.7)	57 (17.8)	21 (14.5)	17 (12.1)	
6	112 (18.5)	69 (21.5)	21 (14.5)	22 (15.6)	
7	82 (13.5)	31 (9.7)	22 (15.2)	29 (20.6)	
8	105 (17.3)	44 (13.7)	34 (23.4)	27 (19.1)	
**Access path length [mm] (SD)**					
Cutis to lesion	83 ±26	82 ±27	85 ±27	83 ±26	0.527
Liver capsule to lesion	43 ±26	44 ±21	41 ±21	43 ±21	0.473
**Obtained probes (SD)**	2.6 ±1.3	2.7 ±1.5	2.5 ±0.9	2.4 ±0.9	0.158
**Radiation exposure (SD)**					
Whole examination [mGycm^2^]	498.3 ±429.5	361.0 ±166.6	411.3 ±517.9	900.3 ±495.6	**0.019**
Biopsy scans [mGycm^2^]	80.1 ±58.0	79.7 ±60.0	88.2 ±56.3	72.6 ±56.4	0.053
Intervention *(i)-images [n]*	34.4 ±24.0	34.6 ±23.8	37.7 ±23.4	30.6 ±24.7	0.182
**Complications**					0.870
Sub-capsular liver bleeding	3 (0.5)	1 (0.3)	1 (0.7)	1 (0.7)	
Fluid edge around liver	2 (0.3)	1 (0.3)	0	1 (0.7)	
Bleeding along the access path	1 (0.2)	0	1 (0.7)	0	
Circulatory problems	1 (0.2)	1 (0.3)	0	0	

Values are mean and standard deviation or number (%)

i.v. = – intravenous; SD = Standard deviation

### Lesion hitting rate

**FIGURE 4.**
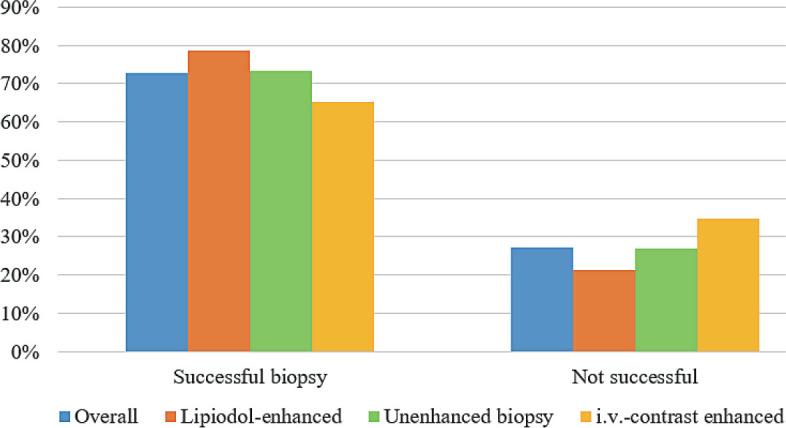
Presentation of histopathologically correlated puncture successes for the overall cohort and individual groups individually.

The target lesion was successfully biopsied in 444 patients (73.1%). In 163 patients (26.9%), the biopsy probe revealed healthy liver tissue defined as non-hitting of the target lesion. For group 1 with unenhanced CT, the biopsy was successful in 73.8% (n = 232). Group 2, with a prior Lipiodol-marking of the target lesion, showed the significantly highest hitting rate with 79.3% (n = 115) (Figure 2). The lowest hitting rate was seen in group 3 with IV contrast medium with 65.2% (n = 92) (Figure 3). The difference between all three groups was stated as significant (p = 0.037). In patients with liver target lesions smaller than 20 mm, the successful hitting rate was significantly higher after Lipiodol-marking compared to unenhanced or intravenous contrast-enhanced images (71.2%, 65.5%, and 47.7%, respectively) (p = 0.021). The location within the liver based on the liver segment model had no impact on the lesion hitting rate (p = 0.107) (Figure 4).

The presence of liver cirrhosis showed no significant impact on the overall success of liver biopsy in all three groups (p = 0.94). In the subset of patients without liver cirrhosis, a successful biopsy was highest in the Lipiodol group, 74.4%, compared to 71.5% in the unenhanced group and 58.5% using IV contrast (p = 0.049).

Patients with a known malignant disease also showed a significant difference comparing the three procedures. A successful biopsy was possible in 83.0% (n = 39) using Lipiodol-marking, in 68.2% (n=131) unenhanced, and in 58.3% (n = 35) with IV contrast medium (p = 0.024). The entity of the biopsied lesion did not impact the hitting rate by showing no significant difference between the protocols.

Based on the performed logistic regression analysis, the lesion diameter (regression coefficient 0.023, p = 0.048) and the number of obtained probes (r = 0.199, p = 0.047) significantly impacted the hitting rate. Age and length of the puncture path, from cutis or liver capsule to lesion, revealed no significant impact.

### Histopathological results

Histopathological results revealed that the major malignant disease was HCC in 155 cases (25.5%). Inflammatory changes in the liver tissue were found to lead the non-malignant changes in 134 patients (22.1%). Appendix 1 shows the high variability of the diagnosed diseases.

### Radiation exposure

Overall DLP was 498.31 mGycm^2^ in mean (± 429.53 mGycm^2^) for the complete examination, including control and post-procedure scan. In the unenhanced group 1, 361.00 mGycm^2^ (± 166.55 mGycm^2^), in the Lipiodol-marking group 2, 411.27 mGycm^2^ (± 517.87 mGycm^2^), and in the IV contrast-medium group 3 900.27 mGycm^2^ (± 495.55 mGycm^2^), reaching a significant difference with higher exposure in group 3 (p = 0.019).

Only counting the applied radiation during the biopsy scans, which contain the scans for needle placement, positioning, and sample acquisition, overall DLP was 80.07 mGycm^2^ in mean (± 57.97 mGycm^2^), in group 1 with 79.69 mGycm^2^ in mean (± 59.98 mGycm^2^), in group 2 with 88.19 mGycm^2^ in mean (± 56.33 mGycm^2^) and group 3 with 72.59 mGycm^2^ in mean (± 56.37 mGycm^2^), showing no significant differences (p = 0.053). The total amount of the intervention (i)-sequences was n = 34.4 (± 24.0), in group 1 n = 34.6 (± 23.8), in group 2 n = 37.7 (± 23.4), and in group 3 n = 30.6 (± 24.7) equal across all groups (p = 0.182) (Table 2).

For the previously performed angiography in group 2 to mark the lesion with Lipiodol, an overall DAP of 10421 cGycm^2^ in mean (± 10277 cGycm^2^) was noted.

### Complications

In 599 patients, no treatment-related complication was stated in the patient's medical record. Eight patients presented with a grade 1 complication: 3 patients with sub-capsular bleeding (0.5%), minimal hemorrhagic fluid edge surrounding the liver in 2 patients (0.3%), bleeding in the access path (1 patient, 0.2%), bleeding in the tumor (1 patient, 0.2%), and circulatory problems due to orthostatic problems (1 patient, 0.2%). No complications of grade 2 or higher were seen related to the intervention. No significant differences in the occurrence of complications were reported (p = 0.870). Details are given in Table 2.

## Discussion

This study's purpose was to compare three different enhancement protocols, unenhanced, with a prior Lipiodol marking and application of IV contrast, for CT-guided biopsies of unspecified suspect intrahepatic lesions.

CT-guided biopsies of suspect and unclear hepatic lesions are an established procedure in the diagnostic cascade with high reliability and a detection rate based on the study of Haage *et al.* of 90.5%.^21^ Several technical adaptions to increase the safety and success rate are possible, for example, variation of the access path or the needle thickness.^22^ The histopathological reliability of the obtained tissue is very good, as various studies showed with an accuracy between 80% and 97%.^23,24^

A previous study by Gerhards *et al*. investigated the use of Lipiodol for biopsy of HCC-suspect or proven HCC lesions in a very small and selected patient cohort. The lesion hitting rates showed comparable results to our study, with a benefit of Lipiodol confirming our presented results. This study goes beyond the results of Gerhards *et al*. by investigating various lesion entities, different biopsy groups, with and without contrast enhancement, and a larger study cohort.^25^

The detectability of HCC lesions by the additional use of Lipiodol compared to conventional contrast medium was proven by Rizvi *et al*. With prior Lipiodol-marking, the detection rate of HCC lesions was perfect (sensitivity 1.0, specificity 0.6, accuracy 0.91), while conventional contrast medium (sensitivity 0.65, specificity 1.0, accuracy 0.68) showed benefits in discriminating the lesion entity.^26^ Setting this fact in context to our study, only a reliable lesion marking is essential for a successful CT-guided biopsy. This is the case in most lesions and is supported by our results with a favorable lesion hitting rate due to the usage of Lipiodol. A clear delineation and diagnosis of the lesion entity are optional, as it will be diagnosed based on the histopathological probe. Nevertheless, Lipiodol distribution within the suspected lesion can unveil HCCs, which may direct the patient directly into therapy, avoiding a biopsy.^12,17^

We also investigated the impact of existing liver cirrhosis on the biopsy results. Cirrhotic liver might have been a potential influencing factor making a successful biopsy more difficult as the lesion is camouflaged by reticular liver tissue. This was refuted by the results of our study showing no difference between the three study groups. Possible reasons for the results are the stronger tissue response during the intervention allowing for a bit easier needle control or the better visibility of the lesions in unenhanced imaging based on the altered tissue type, even in reticular liver tissue.

Another important point is the impact of lesion size on technical success. Smaller lesions are much more difficult for a secure biopsy.^27,28^ With a previous Lipiodol marking, especially lesions below 20 mm were easier to detect securely and to puncture successfully. Previous studies mainly investigated only HCC lesions with this question, but our study showed a benefit across all entities. Further, small lesions are defined as very variable in the literature with a size below 20 to 50 mm making the existing data inconclusive and problematic to compare with our results. The sensitivity of Lipiodol-CT for this small lesion ranged between 58% and 98% based on previous studies.^29,30,31,32^ For example, our results should be considered in clinical practice for patients with a small unclear lesion in MRI imaging, which will be difficultly detectable in unenhanced CT. These patients benefit the most from a prior marking, with Lipiodol offering the best reliable biopsy method.

Interestingly, the patient group with an intravenous contrast medium application during the intervention showed the lowest lesion hitting rate. We identified two potential biases causing these results. First, these patients were primarily scheduled for interventions without contrast medium. In the CT scan directly before the examination for the biopsy planning, the lesion was not detectable. Reasons for this might have been a small size or a challenging access path. Then the decision to administer IV contrast to visualize the lesion was reached. Second, the time of visibility is limited, and especially small lesions are challenging to detect after wash-out of the contrast medium. Compared to the unenhanced group, the IV contrast group might contain more technically complex patients. Patients presenting small lesions or a challenging access path in pre-interventional imaging might benefit from a prior Lipiodol-marking of the target lesion. Especially lesions that may be isodense in unenhanced, enhanced CT and obscured in delayed phase CT should be marked before the intervention.

As mentioned, most studies investigate the role of Lipiodol in the context of HCC, and it is known that the best and strongest enhancement is seen for hypervascularized lesions. But based on previous studies on TACE, it is known that Lipiodol shows an enhancement also in a wide variability of other tumors, *e.g.*, breast cancer or colorectal carcinoma, which are often hypo vascularized. Although not all lesions enhance strong and homogeneity, the wall area or the area of altered liver tissue around the lesions shows a typical enhancement that can be used for biopsy guidance. This underlines the potential for clinical use even if the entity of the lesion is not known before the intervention.

Besides CT, CEUS (Contrast-enhanced ultrasound) and cone beam CT (CBCT) with intra-arterial contrast application are useful other imaging modalities to assess suspicious intrahepatic lesions interventionally.^33^ These techniques also allow for the accurate detection and localization of hepatic lesions. However, both modalities may not be widely available in all medical facilities, limiting their accessibility for some patients. Additionally, CEUS requires highly skilled operators with specific training and expertise. The quality and accuracy of the results may depend on the operator's skill and experience. CBCT fusion imaging with ultrasound for transcutaneous hepatic interventions is a new approach with promising first results, but it still requires further investigation.^34^

The overall complication rate was very low, with no major complications. This is according to the prevalence of complications reported in other studies^21,22,23^, stating that a CT-guided biopsy is an approved and secure intervention well established in the clinical practice. The contrast medium did not influence the complication rate, even in challenging lesion locations.

Besides all benefits of the biopsy, the application of Lipiodol is an interventional procedure with the potential occurrence of complications and increased radiation exposure. Although a transarterial embolization is established in the clinical routine, the rate of major complications is low at under 0.9%.^35^ Additionally, the increased radiation exposure comes with the risk of stochastic radiation damage and radiation-related late effects. Consequently, the application should be reserved for patients who benefit the most. Based on the results, prior Lipiodol-marking can be beneficial in patients with a lesion below 20 mm or a potentially challenging access path.

We acknowledge that this retrospective study had several limitations. This study intended to compare different enhancement protocols for CT-guided biopsies of various intrahepatic lesions. Therefore, we used a retrospective design and a relatively small cohort in a few tumor entities. A study including a prospective design and multiple centers, probably with a closer look at selected lesion entities, is required to prove the results. The histopathological results are problematic in a few cases as the pathologists described several patterns—further, the decision process for the enhancement protocol needed to be randomized. The multidisciplinary tumor board made the decision based on several parameters. This was based on pre-interventional imaging, lesion characteristics, age, or the patient's individual preference. This decision might have led to a selection bias. Also, IV enhancement was favored in the early years, while Lipiodol enhancement was performed based on convincing local experience in the later years. For lesions with the pathological finding of necrosis, this might also have been caused by a diffuse necrotic area, which might be considered as a non-successful biopsy. From the pathological results, no differentiation is possible.

In conclusion, the use of Lipiodol as a contrast agent and pre-puncture marking in angiography increases the lesion-hitting rate in CT-guided biopsies of suspect and unclear hepatic lesions significantly, especially for small suspect liver lesions with a diameter below 20 mm and might be used as an alternative to unenhanced imaging or IV contrast enhancement. For lesions that cannot be seen in unenhanced CT, Lipiodol marking is superior to IV marking and should be preferred.
